# Nerve-Sparing in High-Risk Prostate Cancer: Advantages and Pitfalls of Current Strategies and Technologies

**DOI:** 10.3390/cancers18060945

**Published:** 2026-03-13

**Authors:** Daniele Robesti, Pierluigi Russo, Giuseppe Fallara, Fernando Blank, Massimo Valerio, Ashutosh K. Tewari, Francesco Montorsi, Guillaume Ploussard, Nilesh Patil, Alberto Martini

**Affiliations:** 1 Unit of Urology/Division of Oncology, Gianfranco Soldera Prostate Cancer Laboratory, Urological Research Institute, IRCCS San Raffaele Scientific Institute, 20133 Milan, Italy; robesti.daniele@hsr.it (D.R.);; 2 Department of Urology, Fondazione Policlinico A. Gemelli IRCSS, 00168 Rome, Italy; 3 Unit of Urology, ASST Santi Paolo and Carlo, Milan, Italy; 4 Department of Urology, University of Cincinnati, Cincinnati, OH 45267, USA; 5 Department of Urology, Geneva University Hospital, University of Geneva, 1011 Geneva, Switzerland; 6 Department of Urology, Tisch Cancer Institute, Icahn School of Medicine at Mount Sinai, New York, NY 10029, USA; 7 Department of Urology, La Croix du Sud Hospital, 31130 Touoluse, France

**Keywords:** Prostatic Neoplasm, radical prostatectomy, nerve sparing, high risk, technologies, surgical margins

## Abstract

**Background and Objective**: Positive surgical margins (PSMs) remain a major challenge during radical prostatectomy, particularly in patients with high-risk prostate cancer (HR-PCa), where extracapsular extension, multifocal disease, and aggressive tumor biology substantially increase the likelihood of incomplete resection. In this setting, PSMs are strongly associated with early biochemical recurrence and frequently prompt adjuvant or salvage treatments, potentially exposing patients to overtreatment and added morbidity. **Materials and Methods**: To review and critically appraise established and emerging intraoperative technologies for surgical margin assessment during radical prostatectomy, with a specific focus on their potential role and relevance in patients with HR-PCa. **Evidence Acquisition**: A non-systematic literature review was performed using Pubmed, MEDLINE, Web of Science, and Google Scholar, focusing on preoperative, intraoperative ex vivo, and intraoperative in vivo technologies for margin assessment. Emphasis was placed on techniques with potential applicability to HR-PCa, where real-time intraoperative decision-making is particularly consequential. **Evidence Synthesis**: Preoperative tools, including multiparametric MRI, PSMA-PET imaging, and predictive nomograms, aid surgical planning but show limited sensitivity for microscopic extracapsular extension, especially in high-risk disease. Intraoperative frozen section analysis reduces positive surgical margin rates while enabling selective nerve-sparing (defined as a side-specific, risk-adapted preservation strategy); however, its widespread adoption is constrained by substantial logistical and resource requirements, and robust oncological outcome data in high-risk populations remain limited. Novel ex vivo approaches, such as fluorescence confocal microscopy and specimen-based PSMA PET/CT imaging, offer rapid whole-gland or targeted margin assessment with reduced dependency on dedicated pathology workflows. In parallel, emerging in vivo technologies, particularly PSMA-targeted near-infrared-fluorescence-guided surgery, enable real-time detection of residual tumor and facilitate selective re-resection, representing a biology-driven approach that may be especially suited to HR-PCa. **Conclusions**: In high-risk prostate cancer, intraoperative margin assessment technologies may extend beyond functional preservation and play a central role in optimizing oncological radicality and multimodal treatment sequencing. While NeuroSAFE remains the reference standard, PSMA-based ex vivo and in vivo technologies are particularly promising in HR-PCa due to their ability to integrate tumor biology into surgical decision-making. Prospective studies focusing on high-risk-specific oncological and patient-reported outcomes are needed before widespread clinical implementation.

## Simple Summary

High-risk prostate cancer carries a relevant risk of positive surgical margins after radical prostatectomy, which may lead to disease recurrence and the need for additional treatments. At the same time, preserving the neurovascular bundles is crucial to maintain postoperative sexual function, even in selected high-risk patients. This review summarizes current and emerging strategies to assess surgical margins and guide nerve-sparing decisions in high-risk prostate cancer. Preoperative imaging and risk calculators support surgical planning but remain limited in detecting microscopic tumor extension. Intraoperative techniques, such as frozen section analysis, can reduce positive margins and enable selective nerve sparing (defined as a side-specific, risk-adapted preservation strategy), although they are resource-intensive. Novel technologies, including fluorescence confocal microscopy and PSMA-based imaging approaches, offer rapid, biologically driven margin assessment and appear particularly promising in high-risk disease. Integrating preoperative risk stratification with intraoperative margin assessment may improve oncologic outcomes while preserving function, but high-quality prospective studies are still required.

## 1. Introduction

Radical prostatectomy remains a cornerstone of treatment for patients with high-risk prostate cancer (HR-PCa), either as a standalone approach in carefully selected cases or as part of a multimodal treatment strategy. Historically high risk disease has been classified as the presence of any of the following tumor characteristics: (1) ISUP grade 4/5; (2) PSA > 20 ng/mL; (3) ≥cT2c at digital rectal examination; (4) cN+ status at computer tomography scan. In this population, the surgical objective is inherently complex: achieving maximal oncological radicality while minimizing unnecessary functional compromise [1]. This balance is particularly challenging in HR-PCa, where extracapsular extension, multifocal disease, and aggressive tumor biology substantially increase the risk of positive surgical margins (PSMs) [2,3,4].

PSMs are consistently associated with earlier biochemical recurrence and frequently trigger adjuvant or salvage treatments, including radiotherapy and androgen deprivation therapy [5]. In high-risk disease, the prognostic impact of PSMs appears amplified, as these patients already carry a higher baseline risk of progression and cancer-specific mortality [6]. Consequently, the quality of surgical excision plays a critical role not only in local disease control but also in determining subsequent treatment sequencing and the overall burden of therapy [7].

Nerve-sparing radical prostatectomy has traditionally been approached with caution in HR-PCa due to concerns regarding oncological safety [8]. While wide excision may reduce the risk of residual disease, it often results in significant functional impairment, particularly erectile dysfunction, with limited evidence that systematic non-nerve-sparing surgery translates into superior long-term oncological outcomes. This has led to growing interest in selective, anatomy- and risk-adapted nerve-sparing strategies, even in high-risk settings, provided that oncological control can be reliably maintained [9].

In this review, we discuss the rationale for selective nerve-sparing (defined as a side-specific, risk-adapted preservation strategy) in high-risk prostate cancer and provide a stepwise framework that integrates preoperative risk assessment with emerging intraoperative margin-assessment technologies to optimize oncological and functional outcomes.

## 2. Materials and Methods

**Figure 1:**
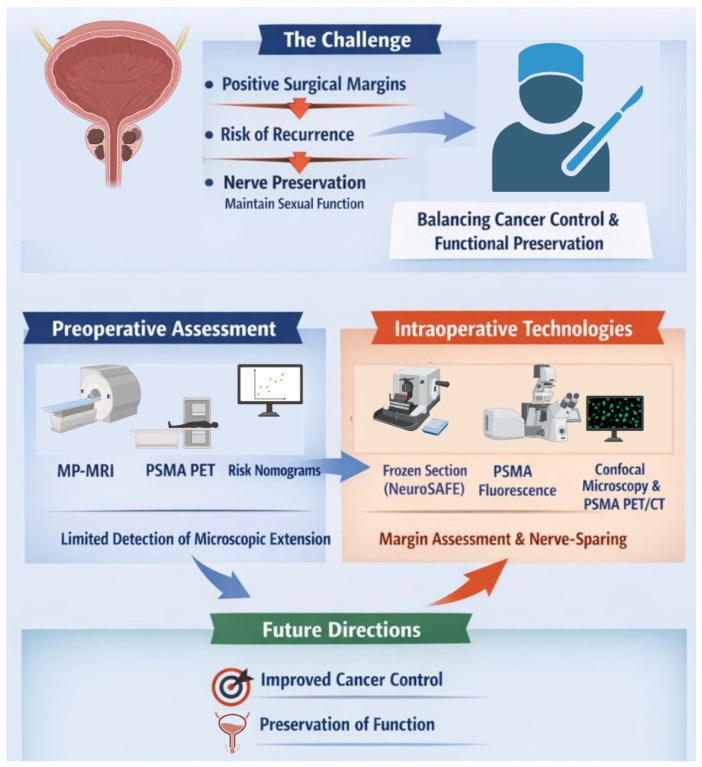
Schematic representation of the topics explored in the review (Created in Biorender. Daniele Robesti 2026. https://app.biorender.com/illustrations/5fbfb98a7a51dc00a390532d?slideId=78554334-5cb2-4be6-990b-6fa2e5675bf0, accessed on 8 March 2026).

This non-systematic narrative review was conducted through searches in PubMed, MEDLINE, Web of Science, Scopus, and Google Scholar using combinations of the following keywords: “frozen section”, “intraoperative”, “real-time”, “prostatectomy”, “margin”, “surgical margin”, “technology”, “imaging”, “fluorescence”, and “nerve sparing” (December 2025). Reference lists of selected articles were manually screened to identify additional relevant studies. The complete search strategy is detailed in the Supplementary Materials section. Given the broad and rapidly evolving landscape of intraoperative technologies, this review does not aim to provide an exhaustive catalog of all available approaches. Instead, the focus was placed on technologies with established clinical relevance, historical significance, or realistic potential for broader implementation in the near future (Figure 1). For conceptual clarity, modern technologies were stratified according to their clinical application stage into: (1) preoperative tools, (2) intraoperative in vivo approaches, and (3) intraoperative ex vivo techniques for surgical margin assessment in high-risk prostate cancer.

## 3. Evidence Synthesis

### 3.1. Pre-Operative Management in High-Risk Prostate Cancer

In patients with high-risk prostate cancer (HR-PCa), pre-operative management plays a pivotal role in defining the initial surgical strategy, particularly with respect to nerve-sparing decisions. In this scenario, the concept of selective nerve sparing refers to a risk-adapted strategy, in which the decision to preserve or resect the neurovascular bundle is made on a side-specific oncological basis, informed by imaging findings, clinical features, and intraoperative assessment. This concept differs from traditional descriptions of partial or incremental nerve sparing, which instead refer to the technical degree of fascial preservation once the decision to spare has already been made. However, unlike low- and intermediate-risk disease, pre-operative assessment in HR-PCa must acknowledge the intrinsic limitations of anatomical imaging and risk models in reliably excluding microscopic extracapsular extension (ECE). As a result, pre-operative tools should be viewed not as definitive decision-makers, but as components of a probabilistic framework guiding selective nerve preservation [10].

#### 3.1.1. Role of Pre-Operative Imaging

Multiparametric magnetic resonance imaging (mpMRI) represents the cornerstone of local staging in contemporary prostate cancer care and is universally recommended prior to radical prostatectomy. In HR-PCa, mpMRI provides valuable information regarding tumor laterality, lesion size, capsular contact, and the presence of overt ECE or seminal vesicle invasion (SVI) [10]. Nevertheless, its sensitivity for microscopic ECE remains suboptimal, with a non-negligible proportion of patients harboring pathologically confirmed ECE despite negative or equivocal imaging findings. This limitation is particularly relevant in HR-PCa, where aggressive histology and multifocal disease increase imaging–pathology discordance [11,12,13].

PSMA-based imaging, while transformative for nodal and metastatic staging, has shown limited incremental value over mpMRI for local ECE detection in the primary setting [14]. Consequently, neither mpMRI nor PSMA-PET alone can safely justify blanket nerve-sparing or systematic wide excision in high-risk patients. Instead, imaging findings must be integrated with pathological and clinical variables to inform side-specific surgical planning [15].

#### 3.1.2. Risk Stratification Beyond Imaging

Recognizing the inadequacy of a “one-size-fits-all” surgical approach in HR-PCa, Martini et al. developed a pragmatic and mpMRI-based algorithm to personalize nerve-sparing decisions in men with unilateral high-risk disease [16]. Using a large, multi-institutional European cohort, the authors specifically addressed the clinically relevant question of when contralateral nerve sparing may be safely considered in the presence of unilateral HR features.

The algorithm focuses on predicting the risk of contralateral ECE, which was observed in approximately 12% of patients overall, despite unilateral high-risk disease on pre-operative assessment. By applying a recursive machine-learning partitioning method (CHAID), three distinct risk categories were identified based on readily available pre-operative variables: (1) Low contralateral ECE risk (~5%): absence of SVI on mpMRI and index lesion diameter ≤15 mm, combined with negative or ISUP 1 contralateral biopsy. (2) Intermediate contralateral ECE risk (~14%): index lesion diameter ≤15 mm with contralateral ISUP 2–3 disease, or index lesion diameter >15 mm with negative or ISUP 1 contralateral biopsy. (3) High contralateral ECE risk (~26%): presence of SVI on mpMRI, or index lesion diameter >15 mm combined with contralateral ISUP 2–3 disease.

Importantly, this algorithm outperformed previously published mpMRI-based side-specific nomograms in predicting contralateral ECE, highlighting the added value of integrating lesion size and biopsy grade with imaging features.

Crucially, the robustness and generalizability of this approach were subsequently confirmed through an independent external validation performed across multiple tertiary referral centers [17,18]. In the validation cohort, contralateral ECE was observed in 18% of patients overall, with event rates of 8%, 17.2%, and 31% across the low-, intermediate-, and high-risk groups, respectively—closely mirroring those reported in the development dataset. Despite a slightly higher baseline prevalence of contralateral ECE, decision curve analysis reaffirmed the algorithm’s net clinical benefit over hypothetical strategies of always or never performing contralateral nerve sparing, thereby supporting its clinical applicability across different practice settings.

From a clinical standpoint, the model supports full nerve sparing in low-risk patients, incremental or graded nerve sparing in intermediate-risk patients, and avoidance of nerve sparing in high-risk patients, thereby challenging the dogma of systematic bilateral wide excision in all HR-PCa cases.

All currently available algorithms and risk calculators guiding nerve-sparing decisions in high-risk prostate cancer are primarily based on mpMRI findings. PSMA PET/CT, despite its lower spatial resolution compared with mpMRI, may offer complementary information by detecting tumor foci that remain occult on conventional anatomical imaging. Emerging reporting frameworks have introduced structured approaches for local staging with PSMA PET/CT, further supporting its potential role in preoperative risk stratification [19,20]. Nevertheless, molecular imaging findings have not yet been systematically incorporated into established surgical algorithms or predictive nomograms for nerve-sparing decision-making.

### 3.2. Role of Intra-Operative Assessment

Intraoperative assessment of surgical margins has emerged as a key adjunct in the management of high-risk disease, offering the possibility of real-time refinement of the surgical strategy. Intraoperative strategies provide immediate feedback with regard to the completeness of excision and an opportunity for additional resection in case of residual tumor detection. Ideal new technologies should be usable in vivo, be fast to use (minutes), be in real time, be easy, and provide an assessment of the entire surface of the prostate, whilst maintaining a high accuracy and without damaging the specimen for successive conventional histopathological examination. Recently, intraoperative frozen section analysis, such as the NeuroSAFE technique, has demonstrated the ability to reduce PSM rates while enabling selective nerve-sparing [21]. However, its widespread adoption remains restricted by substantial logistical, personnel, and infrastructural requirements, and robust oncological outcome data specifically addressing high-risk cohorts are still limited [22].

#### 3.2.1. Intraoperative Frozen Section (NeuroSAFE)

The neurovascular structure–adjacent frozen section examination (NeuroSAFE) is a standardized intraoperative frozen section technique specifically designed to assess surgical margins at the posterolateral aspect of the prostate, where the neurovascular bundles are located and where the majority of nerve-sparing-related positive surgical margins occur [23]. Following radical prostatectomy with an initial intent of nerve preservation, the prostate specimen is extracted and the periprostatic tissue adjacent to the neurovascular bundles is systematically dissected from the base to the apex. These tissue samples are carefully oriented, inked to preserve anatomical landmarks, and rapidly processed by freezing at approximately −20 to −25 °C. Multiple cryostat sections, typically ranging from 10 to 25 blocks per patient, are obtained and stained with hematoxylin and eosin for immediate microscopic evaluation by a dedicated uropathologist [24].

Frozen section positivity is defined by the presence of malignant glands in direct contact with the inked surgical margin. In the event of a positive frozen section, real-time communication with the operating surgeon allows for immediate targeted secondary resection of the ipsilateral neurovascular bundle or adjacent periprostatic tissue, whereas nerve preservation is maintained when frozen sections are negative [25]. This stepwise “preserve-first, verify-second” strategy enables surgeons to push the boundaries of nerve-sparing dissection while retaining oncological control through histologically guided intraoperative decision-making. In experienced centers, the entire NeuroSAFE workflow can be completed within approximately 35–50 min without significantly prolonging operative time, while maintaining high diagnostic accuracy and preserving the integrity of the specimen for definitive whole-mount histopathological analysis.

In high-risk prostate cancer populations, NeuroSAFE has demonstrated particular value by enabling wider initial nerve-sparing without a proportional increase in positive surgical margins. Across contemporary retrospective series that include patients with high-risk features or locally advanced disease, NeuroSAFE has consistently been associated with higher rates of unilateral or bilateral nerve preservation and a reduction in posterolateral margin positivity compared with standard surgical approaches [26]. Notably, in cohorts explicitly reporting high-risk subgroups, bilateral nerve sparing was feasible in up to 60% of high-risk patients undergoing NeuroSAFE-guided surgery, with secondary neurovascular bundle resection required in a minority of cases following positive frozen sections, thereby mitigating margin positivity in anatomically and biologically high-risk regions.

However, despite these encouraging pathological findings, the reporting of oncological outcomes specifically in high-risk populations remains remarkably limited (Table 1). Most available studies focus predominantly on perioperative metrics and margin status, and frequently lack granular stratification on positive margin extents and clinical significance. In addition, follow-up durations are most of the time insufficient to capture meaningful long-term endpoints [15,27,28,29,30,31,32].

To date, only a single multicenter study by Preisser et al. [33] has reported a robust oncological outcome (metastasis-free survival) in a large cohort of high-risk patients. Importantly, this analysis compared nerve-sparing (unilateral and bilateral) to non-nerve-sparing surgery, rather than NeuroSAFE versus standard care, and included patients treated at two institutions, only one of which routinely employed an intraoperative frozen section strategy. Moreover, not all patients undergoing nerve-sparing surgery in that study were managed with NeuroSAFE, precluding any direct attribution of the observed oncological outcomes to the frozen section technique itself. Notably, at multivariable Cox regression analysis adjusting for established confounding factors, nerve-sparing was not an independent predictor of distant metastasis, with no statistically significant association observed for either unilateral nerve sparing (hazard ratio [HR] 1.32, 95% confidence interval [CI] 0.98–1.72; *p* = 0.10) or bilateral nerve sparing (HR 0.92, 95% CI 0.66–1.29; *p* = 0.60) when compared with no nerve preservation. These findings further underscore that the available evidence does not support a detrimental impact of nerve-sparing per se on metastatic outcomes in high-risk disease, while simultaneously highlighting the absence of NeuroSAFE-specific oncological data capable of disentangling the effect of intraoperative frozen section guidance from nerve-sparing strategy alone.

##### Pitfalls of Intraoperative Frozen Section

Despite its ability to increase nerve-sparing rates and reduce posterolateral positive surgical margins, intraoperative frozen-section-guided strategies such as NeuroSAFE are associated with several important limitations. First, when a positive frozen section is identified, the recommended corrective action is a secondary resection of the ipsilateral neurovascular bundle, which represents an anatomical rather than a tumor-targeted intervention. Consequently, it remains uncertain to what extent this secondary resection effectively removes residual tumor tissue as opposed to excising uninvolved periprostatic structures [34]. In the NeuroSAFE PROOF randomized trial published in The Lancet Oncology [21], tumor was identified in the secondary resection specimen in only 14 of 32 cases (44%), indicating that in more than half of patients, the secondary resection did not contain residual cancer despite a positive frozen section. This finding highlights a fundamental limitation of the technique: a positive frozen section does not reliably identify the exact location or extent of residual tumor. As a result, the corrective action is not tumor-targeted but anatomical, prompting an automatic conversion to an extrafascial resection of the entire ipsilateral neurovascular bundle, which may lead to overtreatment when residual cancer is absent or limited.

Second, the functional consequences of secondary resection remain incompletely characterized [34]. While NeuroSAFE overall improves postoperative erectile function by enabling more frequent nerve sparing, the act of secondary resection itself may partially negate these benefits at the individual level. In a dedicated prospective analysis evaluating unilateral nerve-sparing procedures, patients who required secondary resection following a positive frozen section experienced significantly worse postoperative sexual function scores compared with those who achieved successful unilateral nerve sparing without resection, although outcomes remained superior to those observed after primary non-nerve-sparing surgery. These data suggest that secondary resection represents a functional compromise rather than a neutral corrective maneuver and underscore that nerve-sparing converted intraoperatively is not equivalent to uninterrupted nerve preservation.

Collectively, these limitations emphasize that while intraoperative frozen section serves as a valuable safety net for selective nerve sparing, its corrective component (secondary resection) introduces oncological and functional trade-offs that warrant careful patient selection, standardized protocols, and further investigation, particularly in high-risk prostate cancer populations.

#### 3.2.2. Ex Vivo Fluorescence Confocal Microscopy

Fluorescence confocal microscopy (FCM) is an ex vivo optical imaging technique that generates high-resolution, histology-like images of fresh, unprocessed tissue by combining fluorescent nuclear staining with confocal laser scanning. Following brief immersion of the surgical specimen in a fluorescent dye (such as acridine orange) cellular and architectural features are visualized in real time through en face scanning of the tissue surface. Unlike conventional frozen section analysis, FCM does not require tissue cutting, embedding, or cryostat sectioning; instead, optical sectioning is achieved by rejecting out-of-focus light through the confocal aperture, allowing microscopic evaluation of intact tissue surfaces while preserving specimen integrity for subsequent whole-mount histopathology [35].

Fluorescence confocal microscopy (FCM) [36] has recently emerged as a promising alternative to intraoperative frozen section for real-time surgical margin assessment during radical prostatectomy, with the potential to overcome some of the logistical and resource-related barriers limiting the widespread adoption of NeuroSAFE. Using ex vivo, en face imaging of the prostate specimen, FCM enables rapid, high-resolution visualization of fresh tissue without the need for sectioning, preserving specimen integrity for definitive histopathology and significantly reducing intraoperative turnaround time. Early single-centre and feasibility studies have demonstrated encouraging diagnostic accuracy, with reported sensitivities ranging from approximately 70% to over 90% and specificities consistently exceeding 90% when compared with final paraffin-embedded histology or frozen section analysis. However, these studies are largely heterogeneous, often include low- or intermediate-risk populations, and are primarily designed as diagnostic accuracy or feasibility analyses rather than clinical utility trials.

More robust evidence has recently been provided by the prospective, multicentre IP8-FLUORESCE study [37], which evaluated FCM across the entire prostate surface in a paired and blinded design. While this study confirmed high specificity and reasonable sensitivity, the positive and negative predictive values were strongly influenced by margin prevalence rather than intrinsic test performance of FCM in detecting PSM. In this setting, the relatively low prevalence of positive surgical margins resulted in a high negative predictive value but a more modest positive predictive value, despite favourable specificity estimates (specificity >0.90 in all subgroups considered). This distinction is critical when interpreting FCM performance in clinical practice, particularly in high-risk populations where prevalence, margin length, and anatomical distribution differ substantially from lower-risk cohorts.

Importantly, as summarized in the available literature in Table 2, outcome reporting in high-risk prostate cancer remains extremely limited [38,39]. Only the IP8-FLUORESCE study [37] included a substantial proportion of high-risk patients, reporting a high negative predictive value (0.95) for ruling out positive surgical margins in patients with primary Gleason pattern 4 disease. Nevertheless, none of the available studies evaluated FCM-guided intraoperative decision-making or reported long-term oncological outcomes in high-risk populations. As such, while FCM represents a technically attractive and scalable approach for intraoperative margin assessment, its role in guiding nerve-sparing strategies and its impact on oncological safety in high-risk prostate cancer remain unproven and warrant dedicated prospective utility studies.

##### Pitfalls of Fluorescence Confocal Microscopy

Despite its technical appeal, fluorescence confocal microscopy (FCM) presents several important limitations that currently constrain its clinical applicability. First, FCM does not allow reliable grading of prostate cancer according to contemporary pathological standards. The optical images generated by FCM are optimized for architectural assessment and margin detection but lack the cytological detail required to accurately distinguish Gleason patterns, particularly the nuanced separation between pattern 3 and pattern 4 disease. As a result, FCM is inherently limited to a binary assessment of tumor presence at the margin and cannot provide real-time information on tumor aggressiveness, margin length, or biological relevance.

Second, FCM interpretation requires dedicated training and experience for pathologists, as image appearance differs substantially from conventional hematoxylin–eosin (H&E) histology [40]. FCM images are typically rendered in a pseudo-monochromatic or dual-channel format rather than the familiar multichromatic H&E spectrum, which may initially hinder pattern recognition and increase interobserver variability. In a learning-curve study evaluating 80 fresh prostate biopsies from radical prostatectomy specimens, diagnostic performance was encouraging but clearly experience-dependent [41]. Notably, one of the two readers was a general pathologist rather than a dedicated uropathologist, supporting the potential feasibility of FCM beyond highly specialized centers. At first evaluation, agreement was suboptimal (κ = 0.68–0.79), with sensitivity ranging from 76% to 90% and specificity from 85% to 98%. After a 90-day interval and re-evaluation, agreement improved to an almost perfect level (κ = 0.87 for both raters), overall diagnostic accuracy increased to 95%, and ROC areas reached 0.92–0.93, demonstrating a measurable but relatively short learning curve. These findings suggest that structured training is required, and implementation may remain challenging in low-volume centers.

#### 3.2.3. PSMA-Targed Surgery

PSMA-targeted approaches for intraoperative margin assessment in prostate cancer exploit the high and relatively specific expression of prostate-specific membrane antigen on prostate cancer cells to provide molecular rather than purely anatomical guidance during radical prostatectomy. Three principal strategies have been explored (Table 3).

##### Ex Vivo PSMA PET/CT

Ex vivo PSMA PET/CT of the specimen, most commonly performed using dedicated high-resolution systems such as the XEOS AURA 10 specimen PET/CT, involves preoperative or intraoperative administration of a PSMA-labelled tracer followed by immediate imaging of the excised prostate and/or lymph-node specimens. The AURA 10 system, designed specifically for operating-room use, provides near–microscopic spatial resolution compared with conventional clinical PET/CT, enabling three-dimensional visualization of PSMA-avid tumor foci within the specimen. In high-risk and node-positive (N1) settings, this approach has shown particular promise for ex vivo identification of metastatic lymph nodes and for functional assessment of resection margins, with reported high specificity and negative predictive value. Importantly, specimen PET/CT allows correlation of functional tumor volume with histopathology without interfering with the surgical workflow, although its ability to detect microscopic disease remains limited by tracer resolution and segmentation methodology.

Among the limited evidence available in the high-risk setting, the most relevant data derive from the feasibility study by Oderda et al. [42], who evaluated intraoperative specimen PET/CT imaging using PSMA-labelled tracers during robot-assisted radical prostatectomy. Importantly, only two patients in this series fulfilled high-risk criteria, underscoring the exploratory nature of the available evidence. In this small cohort, ex vivo PET/CT allowed visualization of PSMA-avid tumor foci and intraoperative assessment of surgical margins prior to vesicourethral anastomosis, showing good concordance with final histopathology and correctly identifying negative margins, while yielding inconclusive findings in a locally advanced case. Beyond the initial feasibility experience reported by Oderda et al., additional evidence supporting PSMA-targeted intraoperative margin assessment in high-risk prostate cancer derives from a recent prospective study by Moraitis et al. [43], in which all enrolled patients (n = 7) fulfilled high-risk criteria. In this series, patients received preoperative [^18^F]PSMA-1007 followed by intraoperative ex vivo PET/CT of the prostate specimen using the dedicated XEOS AURA 10 system. A total of 18 intraprostatic lesions were analyzed, and functional tumor volumes were segmented using multiple semiautomatic methods. When compared with whole-mount histopathology, iterative thresholding achieved a sensitivity of 83%, specificity of 100%, positive predictive value of 100%, and negative predictive value of 92% for the detection of positive surgical margins. Notably, five of six histopathologically confirmed positive margins were correctly identified, with PET-based margin diameters strongly correlating with histological measurements. From a clinical standpoint, early follow-up showed biochemical recurrence in one patient and adjuvant radiotherapy in another, while the remaining patients remained recurrence-free at short-term follow-up. Although limited by small sample size and the inability to detect purely microscopic disease due to intrinsic PET spatial resolution, this study represents the largest dedicated high-risk cohort evaluated with specimen PET/CT to date, reinforcing the feasibility and biological plausibility of PSMA-guided margin assessment in aggressive prostate cancer and highlighting the need for prospective trials powered for clinical utility and oncological outcomes.

##### PSMA-Targeted Cerenkov Luminescence Imaging

PSMA-targeted Cerenkov luminescence imaging (CLI) exploits the optical photons emitted by positron-emitting tracers, such as ^68^Ga-PSMA, as they decay within tissue. When applied to the surface of the excised prostate specimen, CLI enables rapid, wide-field visualization of PSMA-avid regions corresponding to tumor involvement at or near the surgical margin. This technique is attractive because it is fast, does not require additional probes, and can image the entire specimen surface. However, optical signal penetration is limited to superficial tissue layers, and false-positive signals may occur due to PSMA expression in benign glands or signal overlap from underlying tumor, restricting its reliability for close or microscopic margins [44].

The largest and most informative clinical experience to date was reported by Darr et al. [44], who conducted a single-centre feasibility study including 10 patients with high-risk primary prostate cancer undergoing radical prostatectomy. In this cohort, three patients (30%) had histopathologically confirmed positive surgical margins (PSMs). On a per-patient basis, CLI correctly identified PSMs in 2 of 3 patients, corresponding to a sensitivity of 67%, while no false-positive margin detection was reported at the patient level, yielding a specificity of 100%. When analysis was performed at the lesion/region-of-interest (ROI) level, a total of 35 ROIs showing elevated CLI signal were evaluated across the prostate surface; 25 of 35 ROIs (72%) were confirmed to contain tumor on standard histopathology, translating into a per-lesion positive predictive value of 72%. Conversely, false-negative findings were observed in one patient with a low-grade (ISUP 1), small (3 mm) positive margin, consistent with lower PSMA expression in less aggressive disease. Importantly, false-positive CLI signals occurred predominantly at the prostate base and were largely attributable to PSMA expression in benign glandular tissue, as confirmed by immunohistochemistry, as well as to signal spill-over from subsurface tumor given the limited penetration depth of Cerenkov photons. Overall, while this study demonstrates the technical feasibility of CLI and its ability to detect clinically significant, PSMA-expressing tumor at or near the resection margin in high-risk patients, its diagnostic performance remains constrained by limited sensitivity for microscopic or low-grade disease and by false positives related to benign PSMA uptake. Moreover, CLI findings were not used to guide intraoperative decision-making, and no oncological outcomes were reported, underscoring that CLI currently remains an investigational adjunct rather than a validated tool for margin-guided surgery in high-risk prostate cancer.

##### Radioguided Surgery

Currently radioguided surgery (RGS) exploits handheld gamma or beta probes to detect radiation emitted from PSMA-labelled tracers intraoperatively, providing real-time auditory or visual feedback to guide tissue resection [42]. RGS has been most extensively validated for nodal staging, particularly in patients with suspected N1 disease, where it can identify small PSMA-positive lymph-node metastases beyond standard dissection templates [45,46]. Its application to surgical margin assessment during primary prostatectomy is conceptually appealing but remains challenging due to limited spatial resolution for superficial margins and difficulty discriminating between true positive margins and close, but negative, resections at the level of the prostate bed [47]. In addition, the cost–effectiveness of radioguided surgery (RGS) has yet to be established, given the significant infrastructural and tracer-related costs relative to its currently unproven incremental oncological benefit.

Beyond current applications, future developments may extend molecular guidance beyond the limits of visible-light imaging. Robotic surgical platforms are already equipped with near-infrared fluorescence capability, routinely used with indocyanine green (ICG) for vascular assessment and lymphatic mapping [48,49,50]. This technological infrastructure opens the possibility of integrating targeted tracers emitting outside the visible spectrum, including PSMA-linked fluorescent or hybrid radiotracers. Rather than relying solely on auditory gamma-probe feedback or relative baseline signal measurements, direct visualization of tracer uptake within the surgical field could enhance spatial orientation and potentially improve sensitivity for detecting residual tumor or involved margins. The integration of molecular imaging with robotic visualization may therefore represent a shift toward a biologically guided surgical approach, allowing surgeons to move beyond purely anatomical cues toward real-time functional assessment.

## 4. Study Limitations

This review has several limitations inherent to its narrative, non-systematic design. Although a structured search strategy was applied and transparently reported, the study selection process was not conducted according to formal systematic review methodology, and therefore residual selection bias cannot be excluded. In addition, much of the available evidence on intraoperative technologies in high-risk prostate cancer is derived from retrospective single-center studies, small case series, and early-phase feasibility analyses. The relative novelty of approaches such as Fluorescence Confocal Microscopy and PSMA-targeted intraoperative strategies further limits the availability of high-level comparative data. Consequently, heterogeneity in patient selection, surgical expertise, outcome definitions, and follow-up duration may affect the generalizability of the reported findings. Finally, the predominance of non-comparative designs precludes robust causal inference and highlights the need for prospective validation studies to better define the oncologic and functional impact of these evolving technologies.

## 5. Conclusions

Selective nerve-sparing radical prostatectomy in high-risk prostate cancer represents one of the most complex decision-making scenarios in contemporary urologic oncology, requiring a careful balance between oncological radicality and functional preservation. Pre-operative imaging and risk stratification tools, including mpMRI, PSMA-PET, and validated side-specific algorithms, provide an essential framework for surgical planning but remain inherently probabilistic and insufficient to reliably exclude microscopic extracapsular disease. In this context, intraoperative margin assessment has emerged as a critical adjunct to refine surgical decisions in real time. Intraoperative margin assessment in high-risk prostate cancer should be viewed not as a single technology-driven solution, but as part of an integrated, stepwise strategy that combines pre-operative risk stratification with selective intraoperative guidance. While NeuroSAFE remains the current reference standard, emerging PSMA-based and optical technologies may complement or refine existing approaches by addressing key unmet needs, including tumor localization and biological characterization. Future research must prioritize prospective, high-risk-specific studies powered for meaningful oncological endpoints, patient-reported functional outcomes, and the need, timing, and duration of adjuvant or salvage multimodal treatment (namely radiotherapy, androgen deprivation therapy, and androgen receptor targeting agents) to define the true clinical value of these technologies and their optimal role in precision prostate cancer surgery.

## Figures and Tables

**Table 1:** Included studies dealing with NeuroSAFE in high risk prostate cancer patients.

Author	Study Type	Number of Patients	Number of High Risk Patients	Outcomes of High Risk Patients
Dinneen et al., 2025	Phase 3 Randomized Clinical Trial	NeuroSAFE Arm, N = 190 Control Arm, N = 191	Na	Na
van der Graaf et al., 2025	Single Center Retrospective Study	NeuroSAFE N = 100	Na	Na
Kroon et al., 2024	Single Center Retrospective Study	NeuroSAFE, N = 962 Control, N = 835	Na	Na
Kinnear et al., 2024	Retrospective analysis of a prospectively maintained database	NeuroSAFE, N = 317 Control, N = 823	NeuroSAFE, N = 143 (45%) Control, N = 279 (34%)	45% of patients in the NeuroSAFE group had high-risk disease, of whom 86/143 (60%) initially received bilateral nerve sparing (with uni- or bilateral neurovascular bundle resection post-frozen section necessary in 13 men)
Ambrosini et al., 2025	Single Center Retrospective Study	NeuroSAFE, N = 1507 * Control, N = 1507 *	Na	Na
Taori et al., 2024	Single Center Retrospective Study	NeuroSAFE, N = 5	0	Na
Köseoğlu et al., 2023	Single Center Retrospective Study	NeuroSAFE, N = 90 Control, N = 118	NeuroSAFE, N = 22 (24%) Control, N = 46 (36%)	Bilateral Nerve Sparing Positive Surgical Margins rate: 1/14, elsewhere PSA persistence: 1/14 Secondary resection Positive Surgical Margins rate: 2/8, at the level of the region of interest of pre-operative imaging Biochemical Recurrence: 1/8
van der Slot et al., 2023	Single Center Retrospective Study	NeuroSAFE, N = 987 Control, N = 47	Compound population, N = 399 (39%)	Na
Gretser et al., 2022	Single Center Retrospective Study	NeuroSAFE, N = 452	Na	Na
van der Slot et al., 2022	Single Center Retrospective Study	NeuroSAFE, N = 959 Control, N = 797	NeuroSAFE, N = 356 (37%) Control, N = 244 (31%)	NeuroSAFE in pT3/4 Overall Positive Surgical Margins: 164 (42%) Clinically insignificant Surgical Margins (≤1 mm, GG3): 30 (8%) Control in pT3/4 Overall Positive Surgical Margins: 147 (51%) Clinically insignificant Surgical Margins (≤1 mm, GG3): Na
Noël et al., 2021	Single Center Retrospective Study	NeuroSAFE, N = 520	NeuroSAFE, N = 255 (49%)	Na
Preisser et al., 2020	Multicentric Retrospective Study	Whole Cohort, N = 4351 **	Whole Cohort, N = 4351 **	No NVB preservation (N= 1143, 26%) Positive Surgical Margins: 683 (60%) Unilateral NVB preservation (N = 1652, 38%) Positive Surgical Margins: 387 (23%) Bilateral NVB preservation (N = 1556, 36%) Positive Surgical Margins 468 (30%) MV Cox regression analysis predicting distant metastasis: Unilateral vs. No NVB preservation HR 1.32, 95% CI: 0.98–1.72, *p* = 0.1 Bilateral vs. No NVB preservation HR 0.92, 95% CI: 0.66–1.29, *p* = 0.6

* After propensity score matching. ** Two-institution series, one of the two used the NeuroSAFE approach.

**Table 2:** Included studies dealing with Ex vivo Fluorescence Confocal Microscopy in high risk prostate cancer patients.

Author	Study Type	Number of Patients	Number of High Risk Patients	Outcomes of High Risk Patients
Rocco et al., 2021	Prospective study	24	5 (21%)	Na
Almeida-Magana et al., 2024	Subgroup experimental analysis of an RCT	31	Na	Na
Musi et al., 2024	Secondary analysis (nonrandomized exploratory endpoint) of a Phase III, monocentric, prospective, randomized trial	45	0	Na
Almeida-Magana et al., 2025	Non-randomised, prospective feasibility study	20	0	Na
Mayor et al., 2025	Multicentre, prospective, and blinded, paired cohort study	156	101 (65%)	Reported 0.95 (0.89–0.98) negative predictive value in ruling out positive surgical margins in Primary Gleason Score ≥ 4 patients

**Table 3:** Included studies dealing with PSMA-targeted surgery in high risk prostate cancer patients.

Author	Study Type	Technology	Number of High Risk Patients	Outcomes of High Risk Patients
Moraitis et al., 2025	Pilot study	Ex vivo PSMA PET/CT	7	Accuracy, sensitive, and specific (up to 94%, 83%, and 100%, respectively) in detecting positive resection margins
Oderda et al., 2023	Case series	Ex vivo PSME PET/CT + PSMA RGS	2	Safety and feasibility evaluation
Darr et al., 2020	Prospective study	8Ga-PSMA Cerenkov Luminescence	10	Safety and feasibility evaluation. CLI correctly identified PSMs in 2 of 3 patients, corresponding to a sensitivity of 67%, while no false-positive margin detection was reported at the patient level, yielding a specificity of 100%.

## Data Availability

No new data were created or analyzed in this study. Data sharing is not applicable to this article.

## Supplementary Materials

The following supporting information can be downloaded at: https://www.mdpi.com/article/10.3390/cancers18060945/s1. Supplementary S1: Supplementary materials and methods.
