# Supplementary material for: Nerve-Sparing in High-Risk Prostate Cancer: Advantages and Pitfalls of Current Strategies and Technologies
Source: Cancers (Basel). 2026 Mar 13;18(6):945. doi: 10.3390/cancers18060945 (PMC13025973; doi:10.3390/cancers18060945)
Supplement: Supplementary file 1 [file cancers-18-00945-s001.zip › cancers-4160909-supplementary/cancers-4160909-supplementary.pdf]

SUPPLEMENTARY MATERIALS AND METHODS

Search Design

The literature search was primarily performed in PubMed. Additional relevant articles were identified through manual cross-referencing of selected papers.

The search strategy was structured around four core thematic domains:

1. High-risk prostate cancer and nerve-sparing surgery
2. Preoperative prediction of extracapsular extension (mpMRI and nomograms)
3. Intraoperative frozen section techniques (NeuroSAFE)
4. Emerging intraoperative imaging technologies (Fluorescence Confocal Microscopy and PSMA-targeted approaches)

Both Medical Subject Headings (MeSH) and free-text terms were combined using Boolean operators (AND/OR) to ensure adequate sensitivity and specificity of the search.

Core Search Strings

The PubMed search strings included:

String#1: High-risk prostate cancer and nerve sparing:

("Prostatic Neoplasms"[Mesh] OR "prostate cancer"[tiab])

AND

("high-risk"[tiab] OR "high risk"[tiab] OR "locally advanced"[tiab] OR "Gleason score ≥8"[tiab] OR "ISUP grade group 4"[tiab] OR "ISUP grade group 5"[tiab])

AND

("nerve sparing"[tiab] OR "nerve-sparing"[tiab] OR "neurovascular bundle"[tiab])

String#2: Prediction of extracapsular extension:

("prostate cancer"[tiab])

AND

("extracapsular extension"[tiab] OR "extraprostatic extension"[tiab])

AND

("magnetic resonance imaging"[Mesh] OR "mpMRI"[tiab] OR "multiparametric MRI"[tiab])

AND

("nomogram"[tiab] OR "predictive model"[tiab] OR "risk calculator"[tiab])

String#3: NeuroSAFE:

("prostate cancer"[tiab])

AND

("NeuroSAFE"[tiab] OR "intraoperative frozen section"[tiab] OR "intraoperative margin assessment"[tiab])

AND

("radical prostatectomy"[tiab])

String#4 Fluorescence Confocal Microscopy / confocal (FCM) (suggested string):

("prostate cancer"[tiab])

AND ("fluorescence confocal microscopy"[tiab] OR "confocal microscopy"[tiab] OR "FCM"[tiab])

AND ("intraoperative"[tiab] OR "margin"[tiab] OR "frozen section"[tiab] OR "radical prostatectomy"[tiab])

String#5 PSMA-guided and fluorescence-based techniques:

("prostate cancer"[tiab])

AND

("PSMA"[tiab] OR "prostate-specific membrane antigen"[tiab])

AND

("radioguided surgery"[tiab] OR "fluorescence-guided surgery"[tiab] OR "ex vivo PSMA PET"[tiab] OR "PSMA fluorescence"[tiab])

Eligibility Criteria

Studies were considered eligible if they:

- Addressed nerve-sparing strategies in high-risk or locally advanced prostate cancer
- Evaluated predictive tools for extracapsular extension
- Investigated intraoperative margin assessment techniques
- Explored emerging PSMA-targeted or optical guidance technologies

Only English-language studies involving human subjects were included. No formal temporal restriction was applied; however, emphasis was placed on contemporary literature reflecting current surgical practice.

Study Selection Approach

Given the narrative nature of this review, studies were selected based on:

- Relevance to the conceptual framework of the manuscript
- Methodological robustness
- Clinical applicability

- Contribution to technological evolution in nerve-sparing strategies

This review was not designed as a systematic review or meta-analysis; therefore, a formal PRISMA flow diagram and structured risk-of-bias assessment were not performed.

In addition, many of the technologies discussed (Fluorescence Confocal Microscopy, and PSMA-targeted intraoperative approaches) are relatively recent innovations. As a consequence, the currently available literature is characterized by a predominance of single-arm studies, small case series, feasibility analyses, and early-phase clinical investigations. Comparative or randomized data remain scarce. Given this context, a formal structured risk-of-bias assessment (e.g., ROBINS-I) was not performed. The ROBINS-I tool is specifically designed for non-randomized studies of interventions that include a comparator group, and its application to single-arm or purely exploratory studies would be methodologically inappropriate.

Instead, methodological limitations, including retrospective design, selection bias, heterogeneity in surgical expertise, and limited external validity, are critically discussed in the main manuscript to provide a balanced interpretation of the available evidence.

Efforts were made to ensure transparency in the search strategy and to critically appraise the strength and limitations of the included studies.

Search yield

The PubMed search yielded the following records across the predefined thematic domains: 188 records for high-risk prostate cancer and nerve sparing (String 1), 49 records for prediction of extracapsular extension using mpMRI and nomograms (String 2), 57 records for NeuroSAFE and intraoperative frozen section techniques (String 3), 37 records for Fluorescence Confocal Microscopy (String 4), and 116 records for PSMA-targeted intraoperative strategies (String 5).

Given the narrative design of this review, retrieved records were screened for conceptual relevance, methodological robustness, and clinical applicability rather than through a formal systematic selection algorithm. Particular emphasis was placed on studies directly addressing nerve-sparing decision-making in high-risk prostate cancer and on investigations evaluating intraoperative technologies with potential impact on surgical margins.

From the identified literature, the following key studies were selected for detailed discussion:

- 8 studies addressing high-risk nerve-sparing strategies and/or preoperative risk stratification (primarily derived from Strings 1 and 2),
- 12 studies specifically evaluating NeuroSAFE and intraoperative frozen section strategies (String 3),

- 5 studies evaluating Fluorescence Confocal Microscopy (String 4),
- 3 studies addressing PSMA-guided intraoperative strategies with direct surgical applicability (String 5).

To ensure completeness of the search, additional exploratory searches were conducted using Google Scholar, MEDLINE, Scopus, and Web of Science (WoS). No additional eligible studies meeting the predefined thematic scope of this review were identified through these supplementary searches.

Manual cross-referencing of selected articles was additionally performed to ensure inclusion of seminal contributions and highly cited foundational studies relevant to the conceptual framework of the review.
